# Determination of the HER2 amplification status by in situ fluorescent hybridization and concordance with immunohistochemistry for breast cancer samples in Colombia

**Published:** 2013-06-30

**Authors:** Adriana Plata, Maria Mercedes Torres, Rocío López, Rafael E Andrade

**Affiliations:** a Departament of Pathology & Laboratories. University Hospital Fundación Santa Fe de Bogotá. Bogotá. Colombia. E-mail: rafael.andrade@fsfb.org.co; b Laboratory of Human Genetics. School of Sciences. Universidad de los Andes. Bogotá, Colombia. E-mail: maritorr@uniandes.edu.co

**Keywords:** FISH, HER2, Immunohistochemistry, breast cancer

## Abstract

**Objectives:**

To determine the status of the HER2 amplification in Breast cancer performed in peripheral laboratories in Colombia by immunohistochemistry and its comparison with central laboratories and the FISH status.

**Methods:**

Four thousand one hundred and five cases referred for the determination of the HER2 status by FISH and/or IHQ to the Department of Pathology of the Fundacion Santa Fe were studied. The analysis included correlation between the IHQ HER2 score submitted by the peripheral laboratory (PL), the HER2 score emitted in the CL and the FISH studies performed in the central laboratory (CL).

**Results:**

Two thousand five hundred and eight HER2 IHQ studies were performed in the (CL), using the Dako Herceptest. With the following results: 68.2 % negative (0-1+); 16,4% indeterminate (2+); 15.3% 3+ and 2.3 % not adequate. 1360/ 1719 cases studied by FISH came from the (PL), and 329 (19.1%) from the (CL). Comparing the IHQ score emitted by the PL and the positive FISH status showed: 6/28 0+ were positive (21. 4%); 7/31 1+ (22. 5%); 397/1240 2+ (32.8%) and 74/91 3+ (81. 3%). In the CL the results were 1/9 0+ (11.1%); 3/18 1+ (16.7%); 154/292 2+ (53%); and 9/9 3+ (100%). Only 1/4 negative cases (0/1+) was in house.

**Conclusion:**

The false negative rate (22%), and false positive results (18.7%), of the HER2 status performed by IHQ in peripheral laboratories in Colombia is unacceptable high as well as the inadequacy of tissue indicating that pre-analytical factors have to be improved in Colombia in order to get optimal results.

## Abstract (es)

**Objetivos:**

Determinar la correlación entre el estado de amplificación del oncogén HER2 en cáncer de seno por inmunohistoquímica y FISH, en laboratorios periféricos y en un laboratorio central de referencia en Colombia.

**Métodos:**

Se estudiaron 4105 casos referidos al Departamento de Patología de la Fundación Santa fe de Bogotá para la determinación del estado de amplificación del HER2 por FISH y/o IHQ. El análisis incluyó la correlación entre los scores del HER2 por IHQ en ambos laboratorios y los estudios de FISH realizados en el laboratorio central.

**Resultados:**

Dos mil quinientos ocho casos fueron estudiados para HER2 por IHQ en el LC (Herceptest de DAKO); 68.2% fueron negativos (score de 0-1+); 16.4% indeterminados (2+) y 15,3% positivos (3+); 2,3% fueron inadecuados. 1360 de 1689 casos estudiados por FISH (80.5%) provenían de LP, y 329 (19.5%) del LC. En los LP se encontró amplificación por FISH en: 6/28 casos catalogados por IHQ como 0+ (21.4%); 7/31 1+ (22.5%) ; 397/1210 2+ (2.8%) y 74/91 3+ (81.3%). En el LC se encontró amplificación por FISH en 1/9 casos catalogados por IHQ como 0+ (11.1%); 3/18 1+ (16.7%), 154/292 2+ (53%); y 10/10 3+ (100%) . Un de los 4 casos falsos negativos fue procesado en su totalidad en el LC.

**Conclusión:**

Los resultados falsos negativos (22.0%) y falsos positivos (18.7%) en el estado del HER2 determinado por IHQ en los LP son inaceptablemente altos en Colombia, indicando se requiere establecer mecanismos estrictos de control de calidad.

## Introduction

Breast cancer (BC) is regarded worldwide as the most common malignancy and is a leading cause of death in women1. Colombia has an incidence rate of 21.5% with a mortality rate of 12.2%^1^. The identification of biomarkers in this disease has significantly contributed to the diagnostic support and treatment of patients. The expression of hormone receptors (estrogens and progestin) and the HER2 oncogene have helped to define biologically distinct subtypes of this pathology^2^. The HER2 oncogene is located in chromosomal region 17q12-21 and is coded as a 187KDa glycoprotein. It is part of the membrane receptors ErBb family with tyrosine kinase activity which is activated through the homo or heterodimenzation with the epidermal growth factor (EGFR) ^3^ ^-^ ^5^.

HER2 has been regarded as an important prognostic and predictive biomarker of the disease and is amplified and/or over-expressed in 20-25% of cases defining a tumor subtype of greater aggressiveness^6^. Additionally, patients with advanced states and HER2 amplification are more resistant to conventional anti-cancer treatments and hormone therapy. However, they do benefit from a combined treatment of anti-neoplastics and trastuzumab (Herceptin, Genentech Inc, South San Francisco, CA), a humanized monoclonal antibody that is directed against the extracellular domain of the receptor blocking the signal transduction cascade that activates HER2^7^.

There are different analytical tests that establish the HER2 amplification status. The main clinical application methods are immunohistochemistry (IHC), polymerase chain reaction (PCR) and fluorescent in situ hybridization (FISH). The latter is considered the most sensitive test, or "gold standard". The PCR reaction is used as part of the studies to identify genetic profiles for the Oncotype and Mamaprint tests^8^ ^-^ ^9^. According to the recommendations of the American Society of Clinical Oncology/College of American Pathologists (ASCO/CAP), testing should be performed to assess HER2 status in any patient with a new diagnosis of infiltrating breast cancer^10^ ^,^ ^11^.

**Figure 1:**
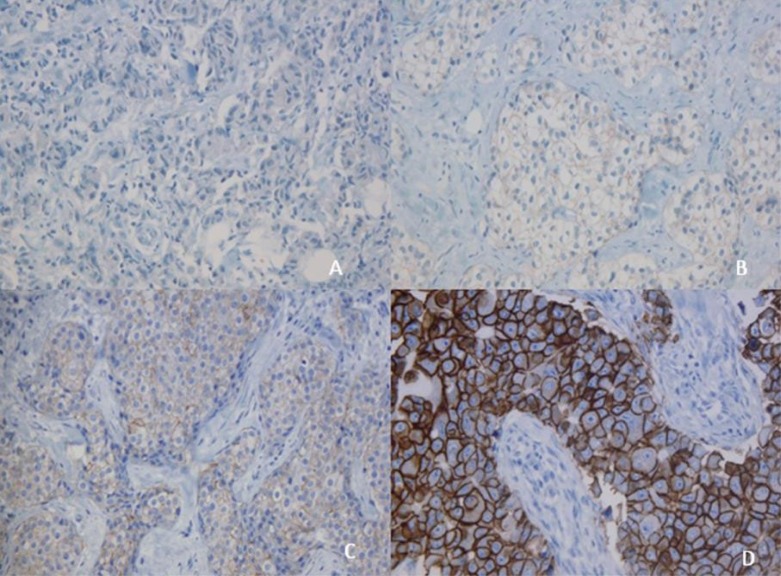
A: Immunohistochemistry studies for detection of oncogene HER2 amplification with negative result (0+) 20X. B: HER2 amplification with a negative result (1+). 20X. C: (2+) HER2 amplification with an "ambiguous" (2+) result. 20X. D: HER2 amplification with a positive result (3+).20X.

IHC is a method that evaluates the HER2 protein expression on the cell surface; a semi-quantitative scale has been used depending on the intensity of reactivity on the membrane and the number of cells observed at light microscopy in different categories (0, 1+, 2+ and 3+). The first two are considered negative, 2+ is considered ambiguous or indeterminate and 3+ is considered positive (Fig.1) 

From the point of view of therapeutic decision making, positive and negative results do not require additional confirmatory studies while the 2+ results do require a confirmatory study by FISH. From a technical perspective, the IHC is an inexpensive test that is performed in most pathology laboratories; however, it has the disadvantage in that normal tissues do not express this protein. Therefore, a positive control does not exist in the tissue to evaluate whether the technique or tissue conditions have been the most appropriate. On the other hand, the interpretation of results is subjective and depends on the training of the observer. This situation leads to the possibility that a significant number of false negatives or positives exist^12^ ^,^ ^13^.

**Figure 2:**
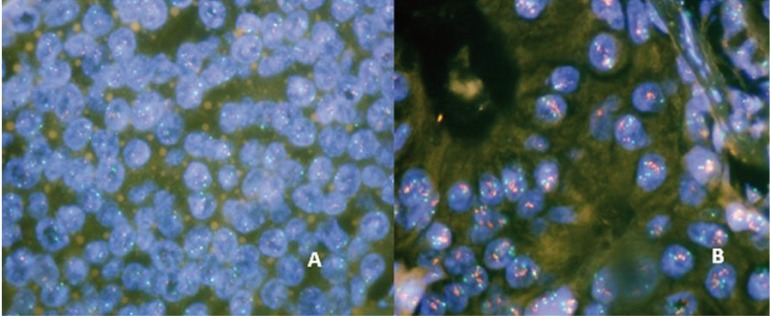
A: Status of oncogene HER2 amplification by the FISH method with a negative result. 100X B: Status of oncogene HER2 amplification by the FISH method with positive result. 100X.

Conversely, the FISH methodology allows evaluation of the state of amplification of HER2, it has greater sensitivity and specificity, and it is based on the application of a DNA probe that hybridizes with HER2, as evidenced by a fluorescent signal. Since all cells contain the genes for HER2, and chromosome 17, it is possible to carry out a simultaneous evaluation process for the quality of nucleic acid preservation (internal tissue control). In cases where there is an amplification of the oncogene, an increase in the number of signals corresponding to HER2 is identified and an increase in relation to the signals of the gene and the centromeric signals of cromosome 17^14^ (Fig. 2). This methodology requires a fluorescence microscope and additional equipment which makes it more expensive and therefore restricted to specialized laboratories.

Taking these factors into account, it is expected that a high level of agreement exists between the results obtained with these two methods; it is estimated that this value must be greater than 95%. According to recent studies, this percentage of agreement is usually only obtained in central reference laboratories (CL) in which a large number of samples are analyzed. On the other hand, there are relatively few studies from differing countries that have assessed the level of concordance between peripheral laboratories (PL) and CL. In addition, we do not know of a systematic study being done with regard to this^15^ ^-^ ^19^.

Given that the state of HER2 oncogene amplification has important clinical and therapeutic implications for a condition as prevalent as breast cancer, the purpose of this study was to evaluate the status of HER2 oncogene amplification by IHC and FISH in samples from Colombian patients. Additionally, the purpose was to evaluate the level of agreement or concordance between positive and negative results obtained by IHC between the Department of Pathology and Laboratories of the University Hospital Fundación Santa Fe de Bogota (CL) and PL from different parts of the country.

##  Materials and Methods 

The information collected in this study is part of a research project approved by the Department of Pathology and Laboratories (DPL) and by the ethics committee of the University Hospital Fundación Santa Fe de Bogotá (HUFSFB).

The databases and pathology reports for 4105 consecutive cases of invasive BC referred to the Department of Pathology and Laboratories of HUFSFB between 2004 and 2010 to evaluate the status of HER2 oncogene amplification by Immunohistochemistry (IHC) and/or fluorescence in situ hybridization (FISH) were reviewed. The origin of cases in part corresponds to samples sent to the PL for confirmatory FISH studies for which the oncogene HER2 status by IHC were only known by the pathology report of the institutions, but the type of test used was unknown. The other group of cases corresponds to samples in which the HER2 by IHC studies were performed in the CL either because they were sent in paraffin blocks and processed by independent laboratories or directly received as formalin fixed material at the Hospital.

**Figure 3:**
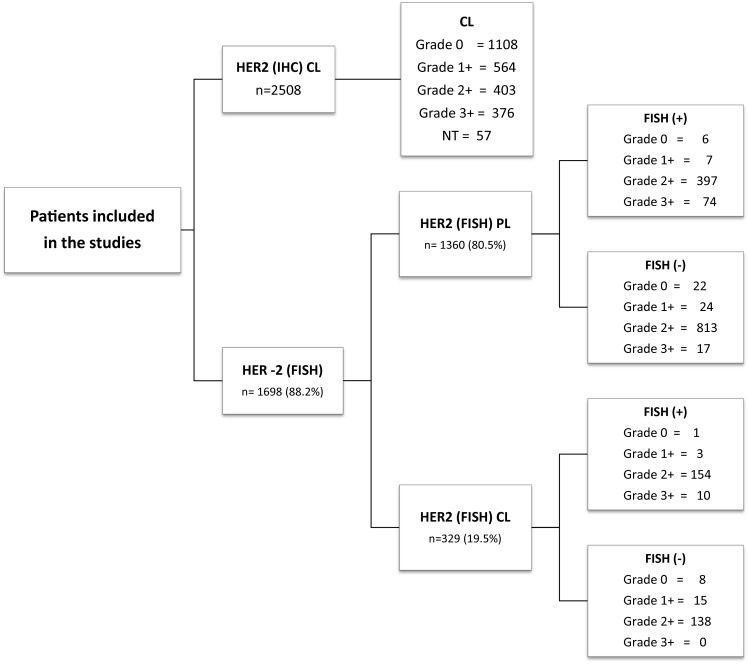
Distribution of cases included in the study

Distribution of cases in the study is shown in Figure 3. All samples were reviewed by two pathologists to confirm the diagnosis of invasive BC. The vast majority of cases referred by the PL correspond to samples in which the result of IHC was ambiguous (2+) and were sent to the CL for the confirmatory FISH study. For studies conducted at the CL, 4µm sections obtained from the paraffin blocks were mounted on electrically charged plates and analyzed by IHC using the Herceptest methodology (Dako K5204) following the manufacturer's instructions. The interpretation of the results was performed following the guidelines recommended by ASCO/CAP^11^ ^,^ ^12^.

For the FISH test 4µm sections were made. Pretreatment was performed with the Vysis kit (Abbott Molecular 32-801200); hybridization was carried out with the Vysion Path (Vysis, Abbott Molecular, Des Plaines, IL), which contains a dual-color probe for the HER2 (17q11. 2-q12-LSI HER2/neu in the orange spectrum) and the centromere of chromosome 17 (17q11.1-q11.1-CEP17 in the green spectrum). Nuclei were counterstained with DAPI (Abbott Molecular) following the manufacturer's recommendations. The signal count from each of the probes (HER2/CEP17) was recorded for each cell and subsequently the ratio HER2/CEP17 in the cell population was calculated. A ratio of HER2/CEP17 equal to or greater than 2.2 was considered amplified, a ratio of HER2/CEP17 less than 1.8 was considered negative, and a ratio of HER2/CEP17 between 1.8 - 2.2 was considered ambiguous.

For the statistical analysis, in order to determine the agreement between the IHC and FISH tests, cases with 2+ or"ambiguous" rating for the two laboratory groups were excluded. The Kappa index was performed according to the scale proposed by Landis and Koch in which, if the agreement is greater than the agreement due to chance, the value of κ is greater than 0. With this test some ranges are defined in which if the coefficient κ is in a range between 0.00 to 0.21 the agreement will be slight, low if the range is from 0.21-0.40, moderate if the range is from 0.41-0.60, good if the range is 0.61-0.80, and a κ coefficient greater than 0.80 would correspond to almost perfect agreement. All statistical analyses were performed in SPSS for Windows, version 17.0. Disagreement was defined as cases in which a positive or negative result by immunohistochemistry gave a result opposite to the FISH.

## Results

### HER2 oncogene status by Immunohistochemistry

Four thousand one hundred and five cases were referred to the LC for study of diagnostic biomarkers in BC. From these, 2,508 cases were evaluated for the oncogene HER2 status by IHC. The results of this marker showed that 66.7% of cases (1,672) were classified as negative; of these, 44.2% (1108/2,508) were 0+, 22.5% (564/2,508) were 1+, 16.1% (403/2,508) were undetermined 2+; 15% (376/2,508) were 3+ positives and the remaining 2.3% (57/2,508) were inappropriate samples for the study according to the criteria of the ASCO/CAP (2008).

### Correlation between Immunohistochemistry studies for HER2 done in peripheral laboratories and the FISH study

The amplification status of HER2 by FISH was evaluated in 1,915 cases, of which 1,689 (88.2%) produced an adequate hybridization signal. Eighty and one-half percent (80.5%) of the cases in which the study could be performed came from differing PL´s of the country. The HER2 results by IHC were obtained from pathology reports and showed the following distribution: 1210 were classified in the category of "ambiguous" or 2+, in which the amplification of HER2 was found in 32.8% (397) of the cases. In the 150 remaining cases, gene amplification was identified in 13/59 cases previously interpreted as negatives (0 and 1+) and in 74/91 cases whose IHC study was interpreted as 3+ (Table 1).

**Table 1:**
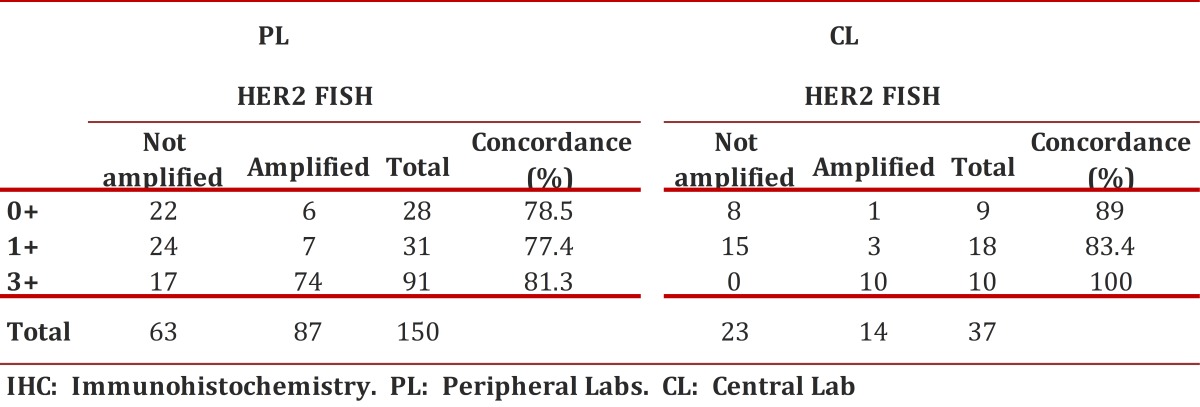
Concordance study to evaluate the amplification status of the oncogene HER2 by the IHC and FISH methods

### Correlation between Immunohistochemistry studies for HER2 and the FISH study performed at the Central Laboratory

Three hundred twenty-nine cases (329) were analyzed in the Central Laboratory corresponding to 19.5% of the total cases referred and in which IHC and FISH studies were performed. The great majority of the FISH studies were performed on samples included in category 2+ (292); the remaining group (37) corresponded to negative or positive cases for which a physician request was received for conducting this test. In 154 cases or 52.7% of those classified as 2+, HER2 amplification was found. In the remaining cases HER2 amplification was identified in 4 of 27 cases that were interpreted by IHC (0-1+) as negative and in 10 cases interpreted as positive (3+) (Table 1).

With respect to the 4 cases that had negative IHC results but showed amplification with FISH, only one was entirely processed in the CL. The 3 remaining cases were initially processed in the PL and paraffin blocks were referred to the CL for performing IHC and FISH studies. In all cases the time of tissue fixation was unknown. As to cases that were given a positive results by the FISH method, the range of amplification given by the ratio HER2/CEP17 ranged from 2.65 to 4.80 for the categories defined by IHC as 0+; 2.2 - 7.5 for 1+; 2.2 to 15.2 for 2+; and 3.5 to 14.5 for the 3+ category.

### Concordance study to assess the status of HER2 oncogene amplification by the IHC and FISH methods

For statistical analysis of concordance, all cases classified as 2+ by IHC were excluded since a confirmatory test by FISH was always performed for these patients. The kappa (k) index was used to assess the HER2 oncogene status by IHC and FISH methodologies, which was globally calculated for 187 cases whose results from the IHC came from both CL and the PL. It yielded an overall k value of 0.63. This result is considered "substantial" on the rating scale. Similarly, concordance studies were carried out on the results obtained independently for each CL and PL. 

The correlation between these two methodologies in the CL was k = 0.76, rated as "substantial" for the PL it was k = 0.59, rated as "moderate" (Table 2). Additionally, a significant statistical association was found between the HER2 oncogene status evaluated by IHC and that evaluated by FISH for samples analyzed in the CL (X2 = 19.04, *p* <0.0001).

**Table 2:**
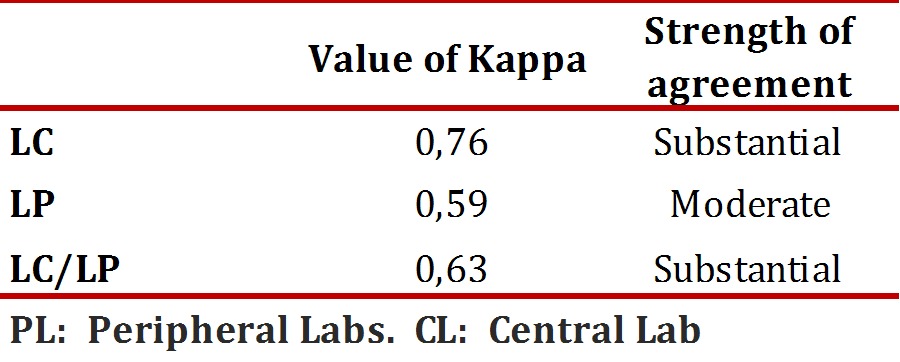
Global concordance index and for each of the laboratories included in the study

## Discussion

Pathology studies play an important role in the evaluation of patients with BC. They have centered on the confirmation of the presence of a tumor, the determination of histologic grade, size, presence of vascular or lymphatic invasion, and the determination of a compromise in the regional lymph nodes. These factors have been fully validated as important prognostic factors and help establish guidelines for determining disease therapeutics. Biomarker studies in BC play an important role in defining predictive factors for response to targeted therapies. The majority of them is carried out by IHC and/or molecular methodologies and is targeted on the expression of hormone receptors (HR), the expression of phenotypic markers of basal cells, cell proliferation markers, such as K167, and the determination of the status of HER2 amplification by IHC or molecular techniques, such as FISH, CISH/SISH, or RT-PCR.

The determination of HER2 status is crucial not only in the definition of an adverse prognostic factor in patients who present with amplification, but in the selection of patients who will possibly have a clinical response to the use of inhibitors for this this oncogene. In this way, the amplification of the oncogene is associated with an increased likelihood of tumor progression, a negative response to chemotherapy, a better response to the use of anthracyclines, a resistance to hormone therapy with tamoxifen, and a response to Trastuzumab^3^ ^,^ ^5^ ^,^ ^7^.

The determination of HER2 status can now be performed by three methods: IHC-based protein expression studies, FISH or CISH colorimetrics, and quantitative PCR studies. Of these, the first two are approved for detection and the latter is used in studies of gene expression profiles included in the Mamaprint and Oncotype regarding the best scheme of therapy to be provided to the pat^8^ ^,^ ^9^. Their correct identification is very important in terms of decision-makingient with BC.

Different groups in the world have shown an important discrepancy in the results of HER2 by IHC when comparing the results between PL that perform a low volume of studies with a reference CL that carries out testing in a routine and systematic fashion. In Canada, O ´Malley *et al.*found false positive rates between 11 and 21% ^20^. In the United States, Roche *et al.* found a concordance rate of 74% 15. Press *et al.* found a 96% rate for negative cases and only a 50% rate with over a thousand patients with 2+ and 3+ ratings.^13^. Perez and colleagues showed a concordance in positive cases close to 80%.^16^Reddy and colleagues showed a percentage of false positives and negatives at 14% and 18%, respectively.^17^ In Greece the results from Papadopulos *et al.* showed concordance of positive cases in only 63% of the sample.^21^ Finally, in Brazil, Wludarski *et al*., in the only Latin American study of which we are aware, reported a concordance for IHC studies of only 34.2% from nearly 150 peripheral laboratories when compared to IHC studies conducted in the CL. Over 70% of cases interpreted as HER2 2+ in the PL were negative in the CL. The majority of positive cases in the CL were considered 2+ in the CL were considered 2+.

In this study, unlike ours, there was no study of concordance between IHC studies performed in the PL and FISH studies performed in the CL, although they noted that the consulting CL had acquired a global concordance percentage of 98.4% with the FISH studies. The latter finding had a false negative rate of 1% for cases rated 0, and 2.7% for the cases rated 1+, along with a percentage of false positives of 1.5% for the cases rated as 3+.

Our study had the objective of evaluating oncogene HER2 status by IHC in the PL and the CL in Colombia, and to compare the results with the amplification status by FISH. The analysis of this study shows that the number of cases with false negative and false positive results carried out by IHC in the PL is unacceptably high in Colombia with a false negative rate of 22.0% and a false positive rate of 18.7%. When we evaluated the number of false negatives in the CL we found that of the 4 cases with 0-1+ ratings that had amplification by FISH, there was only one case where the entire process had been conducted in the CL. The remaining three cases had been processed in the PL and were referred to the CL for IHC and FISH studies.

Thus when we analyzed the cases processed entirely in the CL laboratory there was only one discordant case found and a final concordance index of 0.93 (almost perfect). The overall concordance between positive and negative cases by IHC and the results from FISH was 97.3%. With regard to false negative cases, analysis of the HER2/CEP17 ratio found it equal to 2.4, which although considered positive, is very close to the test cutoff established. In the other three cases, the HER2/CEP ratio ranged from 4.1-7.5. This most likely indicates a poor preservation of antigens in the IHC. The fixation time for the sample was not known in any of these cases. This similarly occurred with the positive cases: 18.7% of patients who were reported as 3+ by IHC in the PL showed no amplification by FISH in the CL, while concordance in the CL between the two methods was 100%.

Within the group of cases interpreted as "ambiguous" or 2+ by the IHC, important differences were also found between the PL and CL laboratories: 32.8% of cases rated 2+ at the PL were found positive by FISH versus 53% reported by the CL. This implies that an important group of studies that really should be negative (0-1+) were reported by the PL as 2+, requiring further confirmatory studies by FISH. In the CL, the ambiguous studies had close to a 50% probability of being positive, reflecting the difficulty of defining with the IHC test the real state of amplification in some cases. These differences are similar to those found in Brazil^18^ and represent an over-interpretation of this category, which has a significant economic impact for health services.

While we were unable to evaluate the IHC studies performed in the PL, the factors recognized as contributing to these differences are most likely of the analytical type related to the interpretation. However, we cannot rule out that there may also be problems with the signal quality.

Finally, we found a very high number of cases (226) or 11.8% of the sample that were referred as 2+ from IHC by the PL and in which it was not possible to recognize an adequate hybridization signal by FISH techniques. This finding corroborates the observations that indicate that the handling of the samples and other pre-analytical factors played a role in the results; particularly in relation to the rate of false negatives. Factors principally contributing to false negative results relate to the conditions of sample handling with the aggravation that it is not possible to have an internal tissue control for IHC studies since normal tissues have undetectable levels of the protein. The prolonged fixation processes, fixing acids or prolonged warm ischemia can lead to degradation of the receptor protein on the one hand or impede its detection by masking related antigens, especially those related to over-fixation in formalin.

Among factors causing false positive results are usually pre-analytical and analytical factors, such as excessive antigen retrieval with exaggerated staining of healthy tissue that should be completely negative or with cytoplasmic staining that impedes the evaluation of staining the cytoplasmic membrane.^10^ ^,^ ^11^ Within analytical factors, the principal cause of false positives is the interpretation of carcinoma areas in situ and the mistaken estimate of the score^11^.

The irregularity and lack of standardization of tissue processes by the pathology laboratories in Colombia not only interfere with protein detection by IHC but with the preservation of nucleic acids essential to achieve adequate FISH results and adequate results from other molecular studies.

This study shows that the peripheral laboratories in Colombia that sporadically perform HER2 studies by IHC are still far from achieving the recommendations made by ASCO/CAP.^10^ ^,^ ^11^ They require concordance values between IHC and FISH of over 95% for both for positive and negative cases.

## Conclusion

Similar to what has been reported in North America, Europe and Brazil, false negative and false positive results in determining the status of HER2 amplification by IHC in the PL are unacceptably high in Colombia with a false negative rate of 22% and a false positive rate of 18.7%. An important number of tissue blocks referred for FISH studies are inadequate, indicating that pre-analytical factors must be improved in Colombia, as in other Latin American countries, to obtain optimal tissues for molecular studies. This is especially needed in a time where directed molecular therapies are tremendously important to the different fields of oncology.Our group has carried out concordance studies by microarrays with a larger number of patients confirming that the concordance of the results in the CL is within the internationally recommended range (data not shown). This confirms that such tests must be performed in reference centers to minimize the number of false positives and negatives.
